# Coronary Artery Ectasia and Aneurysm: Benign Variant or High-Risk Substrate in Need of Tailored Treatment?

**DOI:** 10.3390/jcdd13070336

**Published:** 2026-07-17

**Authors:** Antonios Papoutsakis, Dimitrios Lempidakis, Emmanouil Sideras-Marakas, Eleni Kladou, Stylianos Petousis, Evangelos Zacharis, Georgios Kochiadakis, Emmanuel Skalidis, Michalis Hamilos

**Affiliations:** Cardiology Department, University Hospital of Heraklion, Stavrakia-Voutes, 71100 Heraklion, Greece; antgeopap@yahoo.gr (A.P.); manolis.1994@windowslive.com (E.S.-M.); kl.eleni.92@gmail.com (E.K.);

**Keywords:** coronary aneurysm, coronary ectasia, coronary thrombosis, acute coronary syndrome, percutaneous coronary intervention, treatment

## Abstract

Coronary artery aneurysm (CAA) and ectasia (CAE) are characterized by an abnormal dilation exceeding 1.5 times the reference diameter of the adjacent normal vessel segment. Usually, these vascular anomalies are detected incidentally during coronary computed tomography angiography or invasive coronary angiography. Their clinical significance has become increasingly recognized over time because they may be associated with myocardial ischemia, thrombosis, distal embolization, acute coronary syndromes, and adverse long-term outcomes. In adults, atherosclerosis remains the most frequent cause, while Kawasaki disease is the leading etiology in children. Many patients remain asymptomatic, and the diagnosis is often incidental. Given their variable natural history and poorly delineated prognostic implications, individualized clinical risk stratification is essential. Coronary angiography remains the gold standard for invasive assessment. Management remains controversial in the absence of randomized controlled trials establishing an optimal therapeutic strategy.

## 1. Introduction

Coronary artery aneurysm (CAA) and ectasia (CAE) are characterized by an abnormal dilation over 1.5 times the diameter of the adjacent normal vessel segments, and the angiographic distinction between the two relies on the longitudinal extent of involvement: CAA affects less than 50% of the vessel length, whereas CAE demonstrates a more diffuse dilation [1,2,3,4]. The pathogenesis of coronary aneurysmal and ectatic disease is multifactorial and incompletely elucidated; nevertheless, several mechanistic hypotheses have been proposed. In adults, atherosclerosis remains the most frequent cause, while Kawasaki disease is the leading etiology in children [1,3,4,5]. These lesions are most frequently detected incidentally during non-invasive or invasive coronary angiography. Their clinical significance has garnered increasing recognition over time, as they are associated with myocardial ischemia, in situ thrombosis, distal embolization, acute coronary syndromes, and adverse long-term cardiovascular outcomes [3,4,5,6]. A clinical consensus regarding optimal management remains undefined. Current therapeutic strategies encompass cardiovascular risk factor modification, tailored antithrombotic regimens, percutaneous coronary intervention) PCI for anatomically suitable lesions, and surgical exclusion for giant, complex, or high-risk aneurysms. This reflects the heterogeneity of the disease and the absence of robust comparative data. Coronary aneurysmal disease continues to pose a diagnostic and therapeutic challenge in contemporary cardiovascular practice [1,3,4,5]. This review aims to synthesize current evidence regarding pathophysiology, clinical evaluation, and contemporary management to guide clinical decision-making and highlight areas requiring further research.

## 2. Epidemiology

Epidemiologic data from large angiographic registries reveal a prevalence ranging from 0.3% to 4.9%. In contemporary cohorts isolated CAA occurs at a lower rate of approximately 0.35% [3,4,5,7,8,9,10]. This epidemiologic variability stems largely from heterogeneous diagnostic criteria and detection methodologies across disparate studies. Early investigations such as the Coronary Artery Surgery Study (CASS) registry (comprising patients catheterized between 1975 and 1979) reported prevalence rates up to 4.9%. However, these early cohorts failed to distinguish between focal aneurysmal and diffuse ectatic lesions, frequently including patients with concomitant obstructive disease [1]. In contrast, subsequent single- and multi-center angiographic series utilizing strict, isolated anatomical criteria for focal aneurysms reported substantially lower prevalence rates, ranging from 0.37% to 2.53% [3,4,7,8,11,12]. The most recent data come from the international Coronary Artery Aneurysm Registry (CAAR), which applied prospectively defined criteria for isolated CAA across 436,467 consecutive angiograms from 32 centers (2004–2016), reported a prevalence of 0.35% [4]. Coronary artery ectasia, which by definition is more permissive (diffuse dilation involving a larger proportion of vessel length, frequently coexisting with obstructive disease), is reported separately at 0.85–4.9% depending on whether isolated CAE or CAE with concomitant coronary artery disease (CAD) is counted [5,7,8]. CAA demonstrates a marked male predominance, with male-to-female ratios consistently exceeding 2:1 and reaching up to 3:1 in some series, reflecting potential different sex-specific vascular remodeling [3,4,5,6,13,14,15]. The mean age at diagnosis is approximately 65 years. While atherosclerotic etiologies predominate in adult cohorts, pediatric cases are almost exclusively linked to Kawasaki disease [3,7,11,16,17]. Vessel distribution favors the right coronary artery (RCA), affected in 40–61% of cases, followed by the left anterior descending (LAD) at 15–32% and left circumflex (LCx) at 15–23%. Left main involvement remains uncommon at under 5% [2,3,4,5,7,11,13,14,15,18]. Multi-vessel disease occurs in up to 35% of patients, often correlating with more diffuse Markis Type I or II patterns in which ectasia involves two or more vessels [2,3,4,11,12,13,14,15]. The cardiovascular risk profile mirrors that of atherosclerosis, with prevalent hypertension (45–65%), dyslipidemia (50–90%), and a significant history of smoking (observed in 30–60% of baseline cohorts and up to 70% of acute clinical presentations). Interestingly, observational data consistently suggest an inverse epidemiological association between diabetes mellitus and CAA. It has been hypothesized that diabetic myocardial fibrosis and the consequent downregulation of matrix metalloproteinases may restrict positive outward arterial remodeling. However, this is just an observational correlation rather than a proven pathophysiological mechanism [3,5,7,11,13,14,18,19,20,21,22]. Isolated CAE, in the absence of obstructive stenosis, affects 1–2% of patients undergoing coronary angiograms and carries a risk of adverse events driven by thrombosis rather than ischemia [4,7,8,13,14,15,23]. Long-term follow-up demonstrates elevated rates of major adverse cardiovascular events (approaching 10% annually)-driven predominantly by acute coronary syndromes-although overall survival aligns with matched atherosclerotic cohorts when rigorously adjusted for baseline comorbidities [1,4,13,14,15,18,23].

## 3. Pathogenesis of Coronary Artery Aneurysms and Ectasia

Multiple pathophysiological mechanisms have been proposed to explain this aberrant coronary vascular morphology, which appears to emerge from a complex interplay of destructive and reparative processes. Atherosclerosis represents the predominant etiology in approximately 50% of adult cases. Expansive remodeling initially compensates for plaque accumulation and stenosis, progressing to aneurysmal dilation through proteolysis of the extracellular matrix [1,16,24,25]. Histopathological examination reveals extensive coronary media destruction with lymphocytic infiltration, cystic medial necrosis, and fragmentation of elastic laminae, mediated primarily by matrix metalloproteinases (MMPs) [16,24]. Elevated MMP-2, MMP-3, and MMP-9 activity, accompanied by reduced tissue inhibitors of metalloproteinases (TIMPs) activity, degrades collagen and elastin fibers, weakening the tunica media and predisposing to aneurysmal formation [16,24]. This process is exacerbated by inflammatory cytokines such as interleukin-6 and tumor necrosis factor-α, which upregulate MMP expression in vascular smooth muscle cells and macrophages [16,24]. Enlargement to pathological ectasia occurs when proteolytic imbalance overwhelms structural integrity, particularly under conditions of low endothelial shear stress that promotes atherogenesis and plaque vulnerability. Genetic polymorphisms, including the MMP-3 5A/5A genotype and angiotensin-converting enzyme (ACE) D/D allele, amplify susceptibility by enhancing matrix degradation and vascular remodeling [24]. Beyond atherosclerosis, various inflammatory vasculitides account for 10% to 20% of documented cases. Among these, Kawasaki disease emerges as the predominant pediatric etiology. [16,24,25,26]. Neutrophil-derived serine proteases and mononuclear cell infiltration trigger elastic lamina disruption, while persistent TNF-α signaling sustains MMP activation, yielding aneurysm formation in up to 25% of untreated cases [16,25]. Adult vasculitides such as Takayasu arteritis, polyarteritis nodosa, and systemic lupus erythematosus likewise promote CAA via immune complex deposition and chronic endothelial injury [16,25]. Connective tissue disorders, including Marfan syndrome and Ehlers-Danlos syndrome type IV, predispose individuals to coronary aneurysmal degeneration (often coexisting with aortic root dilation) due to the intrinsic fragility of the tunica media. In these cohorts, fibrillin-1 mutations impair elastic recoil and collagen cross-linking. However, the supporting evidence is derived predominantly from isolated case reports and vascular imaging series rather than dedicated coronary registries [11,16,17,25,27,28,29,30,31,32]. Iatrogenic factors associated with percutaneous coronary intervention, such as target lesion overexpansion, localized hypersensitivity reactions, and delayed reendothelialization following drug-eluting stent implantation, promote aneurysmal dilation (which, in the majority of instances, represents a pseudoaneurysm) in 0.3% to 3.9% of the procedures [16,25,33,34,35,36]. The resulting aneurysmal geometry induces turbulent flow, stasis, and shear stress gradients that propagate endothelial dysfunction and promote thrombus formation with subsequent embolization, accounting for a substantial proportion of ischemic events [16,18,23,24,25,37,38,39,40,41,42]. Stagnant intraluminal blood promotes platelet activation, evidenced by elevated P-selectin and β-thromboglobulin levels, leading to embolization, microvascular obstruction, or acute coronary syndromes [24,25,37]. Giant aneurysms may additionally present an elevated risk of rupture or extrinsic compression of adjacent structures [33,34].

## 4. Clinical Presentation

**Figure 1:**
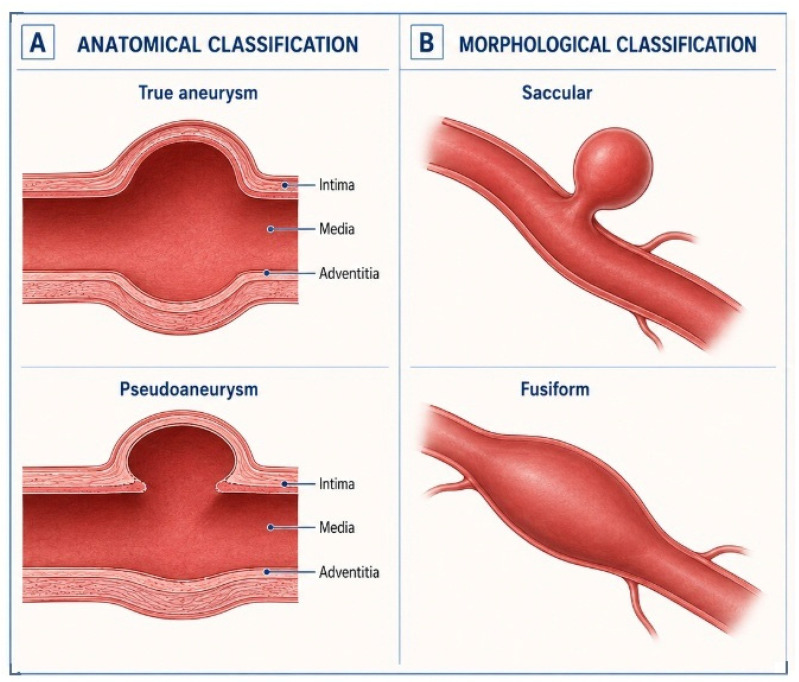
Categorization of coronary aneurysms according to wall composition and morphology/shape.

**Figure 2:**
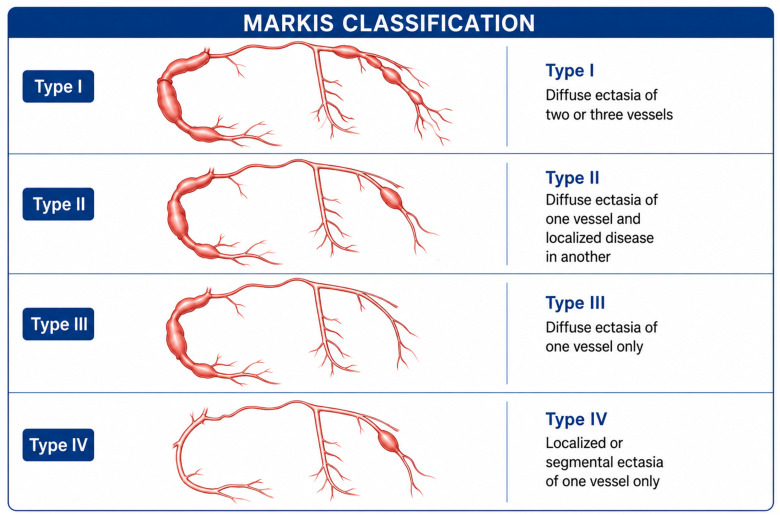
Markis Classification of coronary artery ectasia.

The spectrum of clinical presentations spans from silent disease to life-threatening complications, necessitating a structured diagnostic approach [2,16,33,37]. A dilation exceeding four times the reference coronary artery diameter or presenting with a Z-score greater than 10 in pediatric populations, is clinically classified as a giant CAA. Morphologically, CAAs are categorized into two structural subtypes: saccular aneurysms, where the transverse diameter exceeds the longitudinal extent and fusiform aneurysms, wherein the longitudinal dimension exceeds the transverse, occasionally involving extended or multiple arterial segments (Figure 1). Based on arterial wall composition, lesions are distinguished as true aneurysms, which involve all three architectural layers, versus pseudoaneurysms, wherein layer disruption results in a sac contained merely by the adventitia or perivascular tissue [12,43] (Figure 1). According to the Markis classification which relies on the anatomical extent of the disease, four distinct phenotypes of CAE are identified [2] (Figure 2).

Most patients with CAA/CAE remain asymptomatic, with lesions identified incidentally during invasive coronary angiography or noninvasive coronary computed tomography angiography (CCTA) [4,5,16,37]. When symptoms present, they may be associated with obstructive coronary artery disease (CAD), altered hemodynamics within dilated segments, or thrombotic complications [24]. Stable angina represents the predominant clinical presentation, occurring in up to 60% of identified cases, driven by turbulent hemodynamics within the ectatic segments. The disturbed flow impairs the coronary flow reserve and promotes microvascular dysfunction [17,44,45]. Exercise stress testing frequently unmasks ischemia in these patients, with positive results observed in 40–50% of individuals exhibiting isolated coronary ectasia, even when obstructive coronary artery disease is absent [25,44,45,46]. This phenomenon underscores the importance of functional assessment, as angiographic appearance alone cannot assess the ischemic burden. Within ectatic segments corrected TIMI frame count demonstrates delayed antegrade flow and myocardial blush grade is impaired [11,24,25,47,48]. Acute coronary syndromes (ACS), constitute approximately 20–30% of clinical presentations, frequently complicating proximal aneurysms of the right coronary artery or left anterior descending artery [23]. Acute coronary syndromes are the most serious clinical condition of aneurysmal coronary disease, occurring in 15–30% of diagnosed cases and carrying elevated mortality risk [4,11,16,18,25,38]. Those events arise from thrombus propagation within ectatic segments, distal embolization, or vasospasm superimposed on impaired microvascular perfusion [37]. ST-segment elevation myocardial infarction secondary to aneurysmal or ectatic culprit lesions presents major therapeutic challenges. Primary PCI in this context is associated with significantly higher rates of procedural failure, no-reflow phenomenon, distal embolization, stent thrombosis, and overall mortality compared with interventions in non-aneurysmal vessels. This elevated risk profile is primarily attributable to the heavy thrombus burden and complex lesion morphology [3,5,12,37,38]. In a single-center, retrospective, matched-cohort study by Bogana Shanmugam et al. (25 patients with an ectatic infarct-related artery (EIRA) compared with 80 age, sex, and lesion-matched non-EIRA controls, angiographic success during primary PCI was reported as 24% in EIRA versus 77% in matched non-EIRA culprit vessels. Large thrombus burden was much different between the two groups (96.0% vs. 22.5%, *p* < 0.001) and no stent could be deployed in 44.0% of patients in EIRA group vs. 7.5% in the control group (*p* < 0.001) [49]. At a mean follow-up of 36.6 ± 14.1 months, the EIRA group also had a higher incidence of long-term composite cardiovascular events (44.0% vs. 16.3%, *p* = 0.01) [49]. Given the small sample size of the EIRA group and the single-center, retrospective design, those findings should be considered as hypothesis-generating. In the same line, a recent review and meta-analysis of 10 studies and 13,908 patients with acute myocardial infarction found that angiographic ectasia was associated with significantly higher odds of major adverse cardiovascular events (OR 2.12, 95% CI 1.34–3.36) and recurrent myocardial infarction (OR 2.76, 95% CI 1.62–4.71), but not with all-cause mortality (OR 0.82, 95% CI 0.47–1.43) or cardiac death (OR 1.47, 95% CI 0.81–2.68) [50]. Data from another registry including 2000 STEMI patients confirm that coronary ectasia independently triples the risk of no-reflow (odds ratio 3.2, 95% CI 2.1–4.8) and doubles 30-day mortality, even after adjustment for thrombus aspiration and glycoprotein IIb/IIIa inhibitor use [11,51].

**Figure 3:**
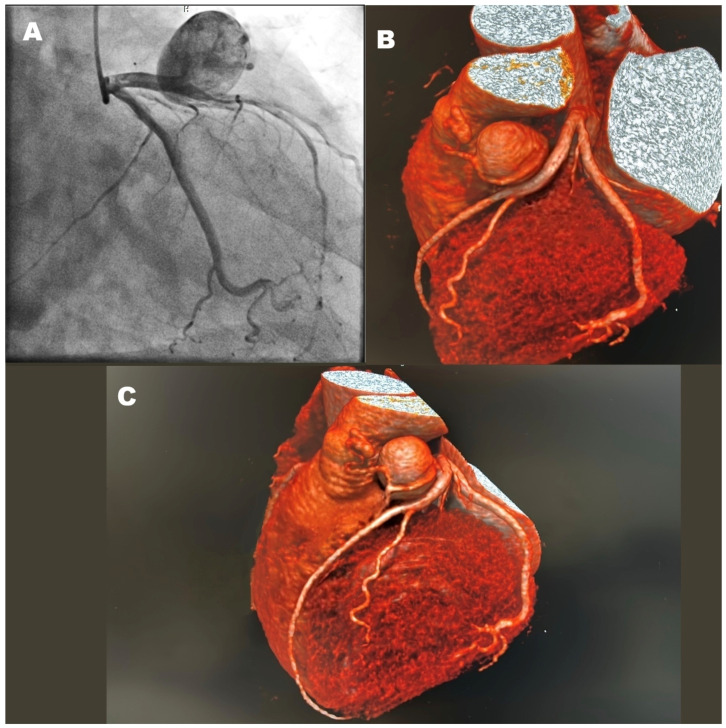
Giant aneurysmatic fistula between the left anterior descending coronary artery and the pulmonary artery (LAD), demonstrated by coronary angiography (**A**) and three-dimensional computed tomography reconstruction viewed from different angles (**B**,**C**).

Rare yet catastrophic manifestations include aneurysm rupture (incidence < 1%), precipitating hemopericardium, cardiac tamponade, or abnormal communication with cardiac chambers [25]. Giant coronary aneurysms may rarely exert a mass effect on adjacent mediastinal or cardiac structures, including the right atrium, right ventricle, or pulmonary artery, and can precipitate congestive heart failure, cardiac tamponade, or, rarely, superior vena cava syndrome [25,33,34]. Although rupture appears to be uncommon, when it happens, it is catastrophic. Rapidly enlarging or symptomatic lesions warrant urgent evaluation with multimodality imaging and consideration of surgical or percutaneous treatment [11,25,33,34,52]. Fistula communication of giant coronary aneurysms to cardiac chambers or great vessels is an uncommon complication and may present with continuous murmurs, myocardial ischemia, or heart failure (Figure 3). Saphenous vein graft aneurysms are rare late complications of CABG, usually detected about 10–15 years after surgery, and may appear as mediastinal masses with compressive physiology, although many of them are discovered incidentally on chest imaging [3,33,34,52,53]. Arrhythmogenic complications, including ventricular tachycardia, have been documented in a subset of patients. These events are potentially linked to myocardial ischemia, microvascular dysfunction, or associated depolarization abnormalities, such as QRS fragmentation [54].

In pediatric populations, Kawasaki disease is the leading cause of coronary artery aneurysms. In the era before intravenous immunoglobulin (IVIG) therapy, 20–25% of untreated patients developed coronary aneurysms, whereas timely IVIG (within the first 10 days of fever onset) reduces this incidence to approximately 4%. IVIG provides systemic immunomodulation via high-dose, pooled donor IgG antibodies, though its precise mechanism of action in Kawasaki disease remains incompletely defined. Proposed pathways include Fc receptor blockade, neutralization of pathogenic antibodies or superantigens, and modulation of cytokine production and regulatory T-cell activity [25,55,56,57,58,59]. Adult inflammatory etiologies encompass Takayasu arteritis, systemic lupus erythematosus, and polyarteritis nodosa, where coronary involvement (up to 54.6% of one cohort) is an independent predictor of cardiovascular events and worse survival [11,16,17,25,27].

## 5. Diagnostic Approach to Coronary Artery Aneurysms and Ectasia

While often detected incidentally during angiography, their recognition demands a systematic diagnostic approach due to associated risks of thrombosis, distal embolization, myocardial ischemia, and rupture. A thorough history should assess cardiovascular risk factors, Kawasaki disease exposure, connective tissue disorders, and prior percutaneous interventions, as atherosclerosis accounts for ~50% of adult cases while inflammatory etiologies predominate in pediatric populations [2]. Physical examination rarely yields specific findings, though signs of systemic vasculitis or Marfanoid habitus may provide diagnostic clues.

### 5.1. First-Line Noninvasive Imaging

Coronary computed tomography angiography (CCTA) serves as the preferred initial modality due to its superior spatial resolution, noninvasive nature, and comprehensive anatomical characterization [48,60]. CCTA accurately delineates aneurysm morphology, size, thrombus burden, wall calcification, and spatial relationships to adjacent structures. Multiplanar reconstructions and volume rendering enable the precise measurement of maximal dimensions and the assessment of complications, such as fistulae or extrinsic compression [33]. Third-generation dual-source scanners minimize radiation exposure (<3 mSv), rendering CCTA suitable for serial surveillance [61]. Transthoracic echocardiography (TTE) complements CCTA by evaluating functional consequences, including regional wall motion abnormalities, ventricular function, and right heart strain from mass effect [1]. Modified parasternal long-axis and apical views often visualize proximal aneurysms adjacent to cardiac chambers. Stress echocardiography (IIa recommendation for Kawasaki patients) detects inducible ischemia from microvascular dysfunction or flow limitation [56].

### 5.2. Invasive Gold Standard: Coronary Angiography

Despite CCTA’s advantages, coronary angiography remains the reference standard for definitive diagnosis and therapeutic decision-making [52]. Characteristic findings include turbulent flow with delayed antegrade filling, segmental backflow, and contrast stasis within dilated segments. Angiography simultaneously identifies concomitant obstructive lesions (present in 50–80% of cases) and guides revascularization [18]. Functional assessment of ischemia is essential for guiding decisions about invasive evaluation or revascularization. When coronary lesions are present, a fractional flow reserve (FFR) of ≤0.80 indicates that revascularization is needed [62]. Alternative functional tests such as coronary flow reserve (CFR), have not been exclusively studied on CAE/CAA. Nevertheless, it is well established that ectatic remodeling inherently impairs microvascular perfusion. Current evidence documents that even in the absence of obstructive epicardial disease, CFR values < 2.0 identify a high-risk phenotype among stable patients. Impaired CFR is associated with approximately two-fold higher rates of all-cause mortality, myocardial infarction, heart failure, and the need for repeat revascularization when compared with patients with preserved CFR [5,63]. There are no data supporting antithrombotic treatment based on low CFR values, beyond standard guideline-based indications [63,64,65,66]. Other invasive indices such as the index of microcirculatory resistance (IMR) have confirmed coronary microvascular dysfunction in ectatic/aneurysmal vessels. Iwańczyk et al. reported significantly elevated IMR without flow-limiting epicardial disease, supporting structural remodeling of the microvasculature, rather than reduction in vasodilator reserve alone, as a key mechanism of ischemia in this setting [67]. Microvascular dysfunction in aneurysmal coronaries can also be identified with the use of myocardial perfusion imaging (SPECT/PET) or stress cardiac magnetic resonance [68].

**Figure 4:**
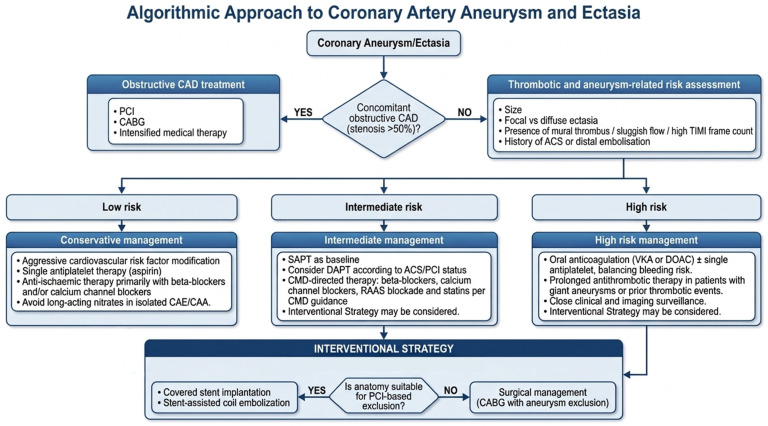
Algorithmic approach to the management of coronary aneurysms.

On the other hand, the role of intravascular imaging in CAA/CAE estimation is very important. When thrombus is present, thrombus burden may underestimate true luminal dimensions. Intravascular ultrasound (IVUS) provides cross-sectional assessment of vessel wall integrity, distinguishing true aneurysms from pseudoaneurysms [69]. IVUS measures precise luminal dimensions for stent sizing, identifies perivascular hematomas, and evaluates stent apposition during percutaneous intervention [70]. Optical coherence tomography (OCT) offers superior resolution (~10 μm) for endothelial characterization and thrombus composition but is limited by shallow tissue penetration (<1 mm) in large aneurysms [24]. Both modalities ultimately enhance procedural safety and optimize long-term outcomes. This structured algorithmic approach, outlined in Figure 4, balances diagnostic accuracy, procedural risk, and radiation dose minimization while facilitating timely clinical intervention. Prospective registries continue to refine thresholds for progression monitoring and therapeutic escalation [71].

## 6. Prognosis of Coronary Artery Aneurysm/Ectasia: Current Evidence

Annual major adverse cardiac event rates approximate 10%, driven primarily by coexisting atherosclerosis rather than aneurysmal complications alone [18]. The American Heart Association (AHA) scientific statement indicates that for medium-sized aneurysms, defined as a Z-score of 5 to less than 10 with an absolute dimension under 8 mm, baseline coronary CCTA may be considered at 1 year, followed by serial surveillance every 2 to 5 years. For large or giant aneurysms, defined as Z score 10 or more or absolute dimension over 8 mm, CCTA may be considered within 2–6 months and then every 1–5 years [72]. The prognosis of coronary artery aneurysm (CAA) and coronary artery ectasia (CAE) is influenced not only by aneurysmal morphology, but also by the burden of concomitant atherosclerotic disease, the percentage of associated coronary stenosis, the left ventricular systolic function, and the clinical presentation. Contemporary observational data indicate that the clinical trajectory of these entities is not uniformly benign. The international Coronary Artery Aneurysm Registry (CAAR) initially included 1565 patients with a median follow-up of 37 months, finding a cumulative major adverse cardiovascular event (MACE) rate of 26.1%. As this ambispective registry expanded over time to include more participating centers and a longer median follow-up of 44.8 months, an updated analysis of 1729 patients demonstrated that MACE occurred in 37.1% and all-cause mortality was 21.9%. Importantly, only 37 patients in this extended follow-up developed local aneurysm-related complications, supporting the concept that adverse outcomes are driven predominantly by the concomitant atherosclerotic burden and clinical risk profile rather than by aneurysmal complications alone [38,73,74,75,76]. This finding aligns with several retrospective data indicating that long-term survival correlates primarily with the extent of obstructive CAD rather than aneurysmal morphology itself [1,2,5,18,37,38,39]. Nonetheless, those observations were made in the context of older angiographic cohorts, where contemporary revascularization strategies and antithrombotic regimens were unavailable. Baman et al. reported that coronary aneurysm was an independent predictor of mortality, with an overall 5-year survival of 71%, supporting the view that aneurysmal coronary disease may pose adverse long-term risk beyond simple anatomical burden of obstructive disease alone [18]. Recent data from the Coronary Artery Ectasia and Aneurysm Registry (CAESAR) evaluated patient outcomes over a median follow-up of 18.9 months, reporting a MACE rate of 38.1%. The presence of a localized aneurysm correlated with a 2.26-fold increase in MACE when compared with diffuse ectasia. This difference was driven predominantly by a five-fold higher risk of non-fatal myocardial infarction. In the CARED-POL registry MACE occurred in up to 10% of these patients annually, due to aneurysm thrombosis leading to artery occlusion or distal embolization [77]. These findings underscore the unique pathophysiological mechanisms driving adverse outcomes in aneurysmal disease, including turbulent blood flow, thrombus formation with distal embolization, and microvascular dysfunction [2,16,18,23,27,37,38,39,40,42]. Thrombotic complications emerge as the dominant prognostic determinant, occurring with greater frequency than in non-aneurysmal CAD [2,16,18,23,27,37,38,39,40,42]. Procedural risks during revascularization constitute another critical prognostic domain. Percutaneous intervention of aneurysmal culprit lesions, particularly during ST-elevation myocardial infarction, carries significantly higher rates of no-reflow phenomenon, distal embolization, and stent thrombosis compared to non-aneurysmal vessels [23,38,42,78]. Meta-analyses report procedural success rates 20–30% lower in CAA/CAE cohorts, with in-hospital mortality approaching 8–10% in thrombotic presentations [23,38,42,78]. In a sex-disaggregated analysis of the CAAR cohort, no statistically significant differences in all-cause mortality or MACE were observed between male and female patients over a median follow-up of 37 months. However, men experienced acute coronary syndromes at a higher frequency than women (15% versus 10%) [79]. In addition, smaller studies suggest that younger patients may exhibit higher inflammatory marker levels and a greater anti-inflammatory response to rosuvastatin [80]. However, evidence that younger age independently predicts accelerated aneurysm progression remains limited [18,23,38,39,40].

## 7. Treatment Strategies for Coronary Artery Aneurysms and Ectasia

CAA and CAE represent complex coronary pathologies requiring individualized therapeutic approaches due to their heterogeneous clinical presentations, anatomical characteristics, and associated thrombotic risk. Therapeutic implications emerge directly from this diverse presentation profile, underscoring the necessity for individualized risk stratification [3,4,11,16,25]. Current management strategies encompass medical treatment, PCI, or surgical revascularization, based on lesion morphology, symptom severity, and coexisting coronary artery disease.

### 7.1. Chronic Coronary Syndromes

In patients with chronic coronary syndromes, the therapeutic priority shifts towards atherosclerosis stabilization and ischemia detection. Asymptomatic patients presenting with small, incidentally discovered aneurysms, without thrombus or ischemia are managed conservatively with aggressive cardiovascular risk factor modification and medical therapy. This includes statins for lipid reduction and plaque stabilization, alongside blood pressure control with the use of renin–angiotensin–aldosterone system (RAAS) inhibitors. These agents may attenuate aneurysmal progression through suppression of matrix metalloproteinase (MMP) activity and inflammatory cytokine production implicated in vessel wall remodeling [16,80,81,82]. For patients with angina or higher-risk anatomical features like diffuse ectasia, large or giant aneurysm, functional assessment with FFR, CFR, invasive microvascular indices or noninvasive imaging studies should precede any revascularization decision [62,63,67]. When revascularization is indicated, the use of intravascular imaging allows anatomical planning, including vessel sizing and side-branch mapping. Procedural success rates in this elective setting are considerably higher than in the acute setting [83,84]. The optimal antithrombotic regimen remains controversial due to limited randomized data. Single antiplatelet therapy (SAPT) (typically low-dose aspirin) is a reasonable strategy for stable patients. This recommendation relies predominantly on expert consensus, pathophysiologic rationale, and extrapolation from Chronic Coronary Syndromes and Kawasaki disease guidelines. Conversely, dual antiplatelet therapy (DAPT) protocols strictly adhere to established guidelines following ACS or PCI [12,65,82,85,86,87,88,89].

Observational data from the Coronary Artery Aneurysm Registry (CAAR) suggest that oral anticoagulation may be associated with fewer coronary ischemic events in patients with coronary artery aneurysm. In a 2:1 propensity-matched cohort (195 OAT vs. 390 non-OAT patients; median follow-up of 3 years), the composite endpoint of myocardial infarction, unstable angina, and aneurysm thrombosis was lower with OAT (8.7% vs. 17.2%; *p* = 0.01), driven by reductions in unstable angina and aneurysm thrombosis, while myocardial infarction and bleeding did not differ significantly. A summary of the antithrombotic treatment strategies is illustrated in Table 1. However, given the non-randomized, nature of the supporting data, the absence of reported adjusted hazard ratios, and the potential for residual confounding, the proposed therapeutic regimens should be treated with caution. Consequently, the current evidence is insufficient to justify the routine administration of oral anticoagulation in this population, thereby necessitating an individualized clinical approach that carefully weighs both thrombotic and bleeding risks. [90,91,92,93].

### 7.2. Acute Coronary Syndromes

Acute coronary syndromes arising from aneurysmal or ectatic culprit lesions pose a fundamentally distinct therapeutic challenge, characterized by a heavy thrombus burden, no-reflow physiology, and important procedural difficulties [49,51,94]. Primary PCI in ectatic infarct-related arteries has been associated with angiographic success rates as low as 24%, no-reflow rates of 13–25%, and in-hospital mortality approaching 8–10% in some series [49,51,94]. Acute management should prioritize aggressive antithrombotic therapy, manual thrombus aspiration and glycoprotein IIb/IIIa inhibitors (where appropriate). Cover stent deployment increases the risk of malapposition and acute stent thrombosis [42,83,95]. When clinically feasible, deferring the interventional treatment of the aneurysmal segment, with aggressive thrombus treatment and the aim of serial intravascular imaging, may be preferable. However, this staged strategy is not supported by robust data and represents an extrapolation from general principles of complex culprit-lesion management in STEMI. Post-procedural DAPT duration follows standard guidelines for ACS/PCI. Consideration of prolonged dual therapy or the addition of oral anticoagulation for large residual aneurysms or documented thrombus, is an option in low bleeding risk [12,85,90].

### 7.3. PCI (Conventional Drug-Eluting Stents/Covered Stents) vs. CABG

The interventional therapeutic strategy selection relies on observational registries, case series, and the anatomical complexity of the concomitant CAD. PCI is favored for single- or double-vessel disease with saccular, focal aneurysms in non–left main segments that offer adequate proximal and distal landing zones for covered stent or coil/plug exclusion, particularly when rapid reperfusion is required in acute coronary syndromes or when surgical risk is prohibitive [4,32,41,81,84,87,96,97,98,99,100]. Covered stents constitute the primary strategy for saccular aneurysms not involving major side branches, with more than 90% angiographic success rate and acceptable long-term patency [83,84]. Conversely, CABG (often combined with aneurysm ligation, exclusion or reconstruction) is generally preferred for giant aneurysms (>4× reference diameter or >20 mm), fusiform or diffuse disease without suitable landing zones, left main involvement, multivessel disease with impaired left ventricular function, or when complications such as extrinsic compression, fistulous communication, rupture risk or mass effect are present, especially in clinically stable patients or after failed PCI. In any case individualized management based on patient’s clinical and anatomic characteristics is of paramount importance [4,32,41,50,81,84,87,96,97,98,99,100].

Covered stent deployment, while theoretically advantageous for aneurysm treatment, introduces additional hazards including malapposition, side-branch compromise, and higher risk for thrombosis, further complicating long-term patency [42,83,95]. The polytetrafluoroethylene-covered GRAFTMASTER (Abbott Vascular) and PK Papyrus (Biotronik) stents demonstrate favorable deliverability profiles [101]. In stent restenosis (ISR), in a single-center cohort of polytetrafluoroethylene covered stents in patients with coronary artery perforation and coronary artery aneurysm, occurred in 25% of lesions, with estimated target lesion revascularization (TLR) rates of 2.6% at 3 years and 17.8% at 5 years. For Papyrus PK stents ISR is estimated between 4–9% after 12–20 months [102,103]. There are no large datasets/trials demonstrating specifically the in-stent restenosis rates for covered stents used exclusively on CAA/CAE [83]. Although, in some reported case- series, drug-eluting stents (DES) were used on top of a covered stent to reduce restenosis, no study has demonstrated that DES over a covered stent reduces restenosis or TLR compared with covered stent alone. [83,101,102,103,104,105]. For fusiform aneurysms or lesions unsuitable for covered stent deployment, stent-assisted coil embolization may be utilized. In this technique, a drug-eluting stent (DES) is deployed across the aneurysmal segment, thereby jailing a previously positioned microcatheter, which facilitates subsequent coil deployment/embolization of the aneurysm [95,101,106,107,108]. Self-expanding stents offer advantages in tapered or tortuous anatomy, adapting to varying luminal diameters while minimizing malapposition [109,110]. Intracoronary imaging can help establish proximal and distal landing zones, which angiography alone cannot reliably delineate in the presence of turbulent flow and contrast stasis [66]. Imaging-derived measurement of the external elastic membrane allows accurate reference-vessel sizing for conventional drug eluting stent or stent-graft diameter selection [69,70]. Both intracoronary imaging modalities characterize thrombus burden and composition within the aneurysmal segment: IVUS detects bulkier mural thrombus owing to its greater penetration depth (5–6 mm), whereas OCT’s superior resolution (~10 µm) better delineates thrombus surface characteristics and underlying plaque morphology, at the cost of reduced penetration (<1–2 mm) [24,67]. Post-deployment imaging confirms adequate stent or stent-graft apposition and expansion [42,83,95].

Coronary artery bypass grafting (CABG) with aneurysmal resection remains the definitive treatment for complex anatomy unsuitable for PCI. [12]. Surgical techniques encompass aneurysmorrhaphy (plication/marsupialization), ligation with bypass, or complete resection with interposition grafting using arterial conduits [111,112]. Hybrid approaches combining PCI with surgical exclusion have emerged for multivessel disease with isolated aneurysmal segments [97,111,112]. Long-term patency exceeds percutaneous strategies, particularly when internal mammary artery grafts bypass proximal aneurysmal disease [113].

## 8. Conclusions

Contemporary epidemiological and angiographic data suggest that CAE and CAA should not be dismissed as benign anatomical variants; conversely, they do not constitute a uniformly malignant clinical phenotype. Instead, they represent a heterogeneous high-risk substrate in which prognosis is dictated by a complex interplay between aneurysm morphology, thrombotic background, microvascular dysfunction, and the burden of concomitant atherosclerotic disease. Over the past decade, diagnostic evaluation of CAE and CAA has shifted from purely angiographic description to a multimodality, physiology-integrated approach. Although modern multimodality imaging offers a detailed assessment of aneurysm morphology, thrombus burden, and spatial relationships to adjacent structures, the optimal timing and frequency of longitudinal surveillance have yet to be established. Regarding therapy, there are no robust clinical data to support the optimal antithrombotic strategy for asymptomatic aneurysms. The comparative role of CABG versus PCI with covered stents remains undefined in the absence of long-term outcome data. This paradigm underscores the unmet need for harmonized definitions and risk classifications, alongside large prospective registries and pragmatic trials, to clarify the natural history of the disease, define optimal medical and antithrombotic therapy, and integrate computational hemodynamics, molecular biomarkers, and microvascular assessment into clinically actionable risk scores.

## Figures and Tables

**Table 1:** Summary of Antithrombotic Strategies in Coronary Artery Aneurysm and Ectasia.

Risk Caregory & Strategy	Indications (Key Features)	Evidence Summary	Uncertainties
High Risk (OAC ± SAPT)	Large/Giant Aneurysm (≥8 mm or Z-score ≥ 10) Documented Mural Thrombus Recurrent ACS or Embolization Extensive Diffuse CAE	Observational (CAAR registry): OAC associated with fewer composite events vs. antiplatelet alone Kawasaki guidelines extrapolation	Lack of RCTs DOACs vs. VKAs efficacy/safety Optimal duration & bleeding risk balance Impact of confounders
Intermediate Risk (Consider DAPT)	Post-ACS Post-PCI Medium Aneurysm/Diffuse Ectasia (without thrombus) Ischemia or CMD present	Standard guideline-directed therapy for ACS/PCI	Benefit/safety of prolonged DAPT (>12 months for unstented disease
Low Risk (SAPT Baseline)	Asymptomatic Small Aneurysm/Limited CAE No Thrombus No prior ischemic events	Standard of Care: Extrapolated from general CAD prevention and pediatric Kawasaki protocols	Impact of SAPT on natural history/progression in isolated disease

OAC—oral anticoagulant, SAPT—single antiplatelet therapy, ACS—acute coronary syndrome, DOAC—direct oral anticoagulant, CAE—coronary artery ectasia, CAAR—Coronary Artery Aneurysm Registry, RCT—randomized controlled trial, VKA—vitamin K antagonists, DAPT—dual antiplatelet therapy, PCI—percutaneous coronary intervention, SAPT—single antiplatelet therapy, CAD—coronary artery disease.

## Data Availability

No new data were created or analyzed in this study. Data sharing is not applicable to this article.

## Abbreviations

The following abbreviations are used in this manuscript:

CAA	Coronary artery aneurysm
CAE	Coronary artery ectasia
RCA	Right Coronary Artery
LAD	Left Anterior Descending Artery
CASS	Coronary Artery Surgery Study
ACE	Angiotensin Converting Enzyme
CCTA	Coronary Computed Tomography Angiography
CAD	Coronary Artery Disease
TIMI	Thrombolysis In Myocardial Infarction
ACS	Acute coronary syndrome
PCI	percutaneous coronary intervention
STEMI	ST-Elevation Myocardial Infarction
CABG	Coronary Artery Bypass Graft Surgery
TTE	Transthoracic Echocardiography
FFR	Fractional Flow Reserve
CFR	Coronary Flow Reserve
SPECT	Single Photon Emission Computed Tomography
PET	Positron Emission Tomography
IVUS	Intravascular Ultrasound
OCT	Optical Coherence Tomography
CAAR	Coronary Artery Aneurysm Registry
MACE	Major Adverse Cardiovascular Events
RAAS	Renin–angiotensin–aldosterone system
SAPT	Single antiplatelet therapy
DAPT	Dual antiplatelet therapy
TLR	Target Lesion Revascularization
LCx	Left Circumflex Artery
MMP	Matrix metalloproteinase
OΜΤ	Optimal medical therapy
IVIG	Intravenous immunoglobulin
EIRA	Ectatic infarct-related artery

## References

[1] Swaye P.S., Fisher L.D., Litwin P., Vignola P.A., Judkins M.P., Kemp H.G., Mudd J.G., Gosselin A.J. (1983). Aneurysmal Coronary Artery Disease. Circulation.

[2] Markis J.E., Joffe C.D., Cohn P.F., Feen D.J., Herman M.V., Gorlin R. (1976). Clinical Significance of Coronary Arterial Ectasia. Am. J. Cardiol..

[3] Sorathia S., Arockiam A.D., Agrawal A., Haroun E., Khurana R., Ahmed A., Cho L., Jaber W., Griffin B., Wang T.K.M. (2025). Contemporary Review of the Clinical Features, Multi-Modality Imaging, and Management of Coronary Artery Aneurysms. Eur. Heart J. Imaging Methods Pract..

[4] Núñez-Gil I.J., Cerrato E., Bollati M., Nombela-Franco L., Terol B., Alfonso-Rodríguez E., Camacho Freire S.J., Villablanca P.A., Amat Santos I.J., de la Torre Hernández J.M. (2020). Coronary Artery Aneurysms, Insights from the International Coronary Artery Aneurysm Registry (CAAR). Int. J. Cardiol..

[5] Hartnell G.G., Parnell B.M., Pridie R.B. (1985). Coronary Artery Ectasia. Its Prevalence and Clinical Significance in 4993 Patients. Br. Heart J..

[6] Almansori M.A., Elsayed H.A. (2015). Coronary Artery Ectasia—A Sample from Saudi Arabia. J. Saudi Heart Assoc..

[7] Devabhaktuni S., Mercedes A., Diep J., Ahsan C. (2016). Coronary Artery Ectasia—A Review of Current Literature. Curr. Cardiol. Rev..

[8] Li Y.-P., Ko C.-Y. (2025). Coronary Artery Aneurysm and Ectasia: A Brief Review. J. Taiwan Soc. Cardiovasc. Interv..

[9] Vadalà G., Di Caccamo L., Alaimo C., Di Fazio L., Ferraiuoli G., Buccheri G., Sucato V., Galassi A.R. (2022). Coronary Arteries Aneurysms: A Case-Based Literature Review. Diagnostics.

[10] Pinar Bermúdez E., López Palop R., Lozano Martínez-Luengas I., Cortés Sánchez R., Carrillo Sáez P., Rodríguez Carreras R., Picó Aracil F., Valdés Chávarri M. (2003). Coronary ectasia: Prevalence, and clinical and angiographic characteristics. Rev. Esp. Cardiol..

[11] Woźniak P., Iwańczyk S., Błaszyk M., Stępień K., Lesiak M., Mularek-Kubzdela T., Araszkiewicz A. (2024). Coronary Artery Aneurysm or Ectasia as a Form of Coronary Artery Remodeling: Etiology, Pathogenesis, Diagnostics, Complications, and Treatment. Biomedicines.

[12] Barioli A., Visco E., Pellizzari N., Marzot F., Lanzellotti D., Favero L., Cernetti C. (2024). Diagnostic Workup and Treatment Options for Aneurysmal Coronary Artery Disease. Vessel. Plus.

[13] Gunes Y., Boztosun B., Yildiz A., Metin Esen A., Saglam M., Bulut M., Karapinar H., Kirma C. (2006). Clinical Profile and Outcome of Coronary Artery Ectasia. Heart.

[14] Harikrishnan S., Sunder K.R., Tharakan J., Titus T., Bhat A., Sivasankaran S., Francis B. (2000). Coronary Artery Ectasia: Angiographic, Clinical Profile and Follow-Up. Indian. Heart J..

[15] Lam C.S.P., Ho K.T. (2004). Coronary Artery Ectasia: A Ten-Year Experience in a Tertiary Hospital in Singapore. Ann. Acad. Med. Singap..

[16] Abou Sherif S., Ozden Tok O., Taşköylü Ö., Goktekin O., Kilic I.D. (2017). Coronary Artery Aneurysms: A Review of the Epidemiology, Pathophysiology, Diagnosis, and Treatment. Front. Cardiovasc. Med..

[17] Richards G.H.C., Hong K.L., Henein M.Y., Hanratty C., Boles U. (2022). Coronary Artery Ectasia: Review of the Non-Atherosclerotic Molecular and Pathophysiologic Concepts. Int. J. Mol. Sci..

[18] Baman T.S., Cole J.H., Devireddy C.M., Sperling L.S. (2004). Risk Factors and Outcomes in Patients with Coronary Artery Aneurysms. Am. J. Cardiol..

[19] Willner N.A., Ehrenberg S., Musallam A., Roguin A. (2020). Coronary Artery Ectasia: Prevalence, Angiographic Characteristics and Clinical Outcome. Open Heart.

[20] Fariba F., Moradi M., Arabi A., Ghaderi B. (2016). Prevalence of Coronary Artery Ectasia with Atherosclerosis and Associated Risk Factors in the West of Iran: A Cross-Sectional Study. J. Res. Health Sci..

[21] Cai Z., Li L., Wang H., Yuan S., Yin D., Song W., Dou K. (2022). Effect of Type 2 Diabetes on Coronary Artery Ectasia: Smaller Lesion Diameter and Shorter Lesion Length but Similar Adverse Cardiovascular Events. Cardiovasc. Diabetol..

[22] Huang Q.-J., Liu J., Chen M.-H., Li J.-J. (2014). Relation of Diabetes to Coronary Artery Ectasia: A Meta-Analysis Study. Anadolu Kardiyol. Derg..

[23] Doi T., Kataoka Y., Noguchi T., Shibata T., Nakashima T., Kawakami S., Nakao K., Fujino M., Nagai T., Kanaya T. (2017). Coronary Artery Ectasia Predicts Future Cardiac Events in Patients with Acute Myocardial Infarction. Arter. Thromb. Vasc. Biol..

[24] Antoniadis A.P., Chatzizisis Y.S., Giannoglou G.D. (2008). Pathogenetic Mechanisms of Coronary Ectasia. Int. J. Cardiol..

[25] Pahlavan P.S., Niroomand F. (2006). Coronary Artery Aneurysm: A Review. Clin. Cardiol..

[26] Cohen P., O’Gara P.T. (2008). Coronary Artery Aneurysms: A Review of the Natural History, Pathophysiology, and Management. Cardiol. Rev..

[27] Syed M., Lesch M. (1997). Coronary Artery Aneurysm: A Review. Prog. Cardiovasc. Dis..

[28] Seeburun S., Wu S., Hemani D., Pham L., Ju D., Xie Y., Kata P., Li L. (2023). Insights into Elastic Fiber Fragmentation: Mechanisms and Treatment of Aortic Aneurysm in Marfan Syndrome. Vasc. Pharmacol..

[29] Moon J.-Y., Lee S.-J., Kang T.S. (2012). The Vascular Aneurysms of Ehlers–Danlos Syndrome Type IV. Eur. Heart J..

[30] Onoda K., Tanaka K., Yuasa U., Shimono T., Shimpo H., Yada I. (2001). Coronary Artery Aneurysm in a Patient with Marfan Syndrome. Ann. Thorac. Surg..

[31] Chu L.C., Johnson P.T., Dietz H.C., Fishman E.K. (2014). CT Angiographic Evaluation of Genetic Vascular Disease: Role in Detection, Staging, and Management of Complex Vascular Pathologic Conditions. Am. J. Roentgenol..

[32] Matta A.G., Yaacoub N., Nader V., Moussallem N., Carrie D., Roncalli J. (2021). Coronary Artery Aneurysm: A Review. World J. Cardiol..

[33] Jeudy J., White C.S., Kligerman S.J., Killam J.L., Burke A.P., Sechrist J.W., Shah A.B., Hossain R., Frazier A.A. (2018). Spectrum of Coronary Artery Aneurysms: From the Radiologic Pathology Archives. Radiographics.

[34] Pham V., Hemptinne Q.d., Grinda J.-M., Duboc D., Varenne O., Picard F. (2020). Giant Coronary Aneurysms, from Diagnosis to Treatment: A Literature Review. Arch. Cardiovasc. Dis..

[35] Aoki J., Kirtane A., Leon M.B., Dangas G. (2008). Coronary Artery Aneurysms After Drug-Eluting Stent Implantation. JACC Cardiovasc. Interv..

[36] Hakeem A., Karmali K., Larue S., Bhatti S., Chilakapati V., Samad Z., Cline M., Cilingiroglu M., Leesar M. (2011). Clinical Presentation and Outcomes of Drug-Eluting Stent-Associated Coronary Aneurysms. EuroIntervention.

[37] Manginas A., Cokkinos D.V. (2006). Coronary Artery Ectasias: Imaging, Functional Assessment and Clinical Implications. Eur. Heart J..

[38] Warisawa T., Naganuma T., Tomizawa N., Fujino Y., Ishiguro H., Tahara S., Kurita N., Nojo T., Nakamura S., Nakamura S. (2015). High Prevalence of Coronary Artery Events and Non-Coronary Events in Patients with Coronary Artery Aneurysm in the Observational Group. Int. J. Cardiol. Heart Vasc..

[39] Demopoulos V.P., Olympios C.D., Fakiolas C.N., Pissimissis E.G., Economides N.M., Adamopoulou E., Foussas S.G., Cokkinos D.V. (1997). The Natural History of Aneurysmal Coronary Artery Disease. Heart.

[40] Gunasekaran P., Stanojevic D., Drees T., Fritzlen J., Haghnegahdar M., McCullough M., Barua R., Mehta A., Hockstad E., Wiley M. (2019). Prognostic Significance, Angiographic Characteristics and Impact of Antithrombotic and Anticoagulant Therapy on Outcomes in High versus Low Grade Coronary Artery Ectasia: A Long-Term Follow-up Study. Catheter. Cardiovasc. Interv..

[41] Núñez-Gil I.J., Nombela-Franco L., Bagur R., Bollati M., Cerrato E., Alfonso E., Liebetrau C., De la Torre Hernandez J.M., Camacho B., Mila R. (2017). Rationale and Design of a Multicenter, International and Collaborative Coronary Artery Aneurysm Registry (CAAR). Clin. Cardiol..

[42] Kawsara A., Núñez Gil I.J., Alqahtani F., Moreland J., Rihal C.S., Alkhouli M. (2018). Management of Coronary Artery Aneurysms. JACC Cardiovasc. Interv..

[43] Dimagli A., Malas J., Chen S., Sandner S., Schwann T., Tatoulis J., Puskas J., Bowdish M.E., Gaudino M. (2024). Coronary Artery Aneurysms, Arteriovenous Malformations, and Spontaneous Dissections—A Review of the Evidence. Ann. Thorac. Surg..

[44] Sayin T., Döven O., Berkalp B., Akyürek O., Güleç S., Oral D. (2001). Exercise-Induced Myocardial Ischemia in Patients with Coronary Artery Ectasia without Obstructive Coronary Artery Disease. Int. J. Cardiol..

[45] Altinbas A., Nazli C., Kinay O., Ergene O., Gedikli O., Ozaydin M., Dogan A., Gunay G. (2004). Predictors of Exercise Induced Myocardial Ischemia in Patients with Isolated Coronary Artery Ectasia. Int. J. Cardiovasc. Imaging.

[46] Krüger D., Stierle U., Herrmann G., Simon R., Sheikhzadeh A. (1999). Exercise-Induced Myocardial Ischemia in Isolated Coronary Artery Ectasias and Aneurysms (“dilated Coronopathy”). J. Am. Coll. Cardiol..

[47] Papadakis M.C., Manginas A., Cotileas P., Demopoulos V., Voudris V., Pavlides G., Foussas S.G., Cokkinos D.V. (2001). Documentation of Slow Coronary Flow by the TIMI Frame Count in Patients with Coronary Ectasia. Am. J. Cardiol..

[48] Mavrogeni S., Markousis-Mavrogenis G., Kolovou G. (2014). Contribution of Cardiovascular Magnetic Resonance in the Evaluation of Coronary Arteries. World J. Cardiol..

[49] Bogana Shanmugam V., Psaltis P.J., Wong D., Meredith I.T., Malaiapan Y., Ahmar W. (2017). Outcomes After Primary Percutaneous Coronary Intervention for ST-Elevation Myocardial Infarction Caused by Ectatic Infarct Related Arteries. Heart Lung Circ..

[50] Otountzidis N., Stalikas N., Baroutidou A., Karagiannidis E., Didagelos M., Fyntanidou B., Ziakas A., Giannakoulas G. (2026). Major Adverse Cardiovascular Events in Patients with Acute Myocardial Infarction and Angiographic Evidence of Coronary Artery Ectasia: A Systematic Review and Meta-Analysis. J. Clin. Med..

[51] Schram H.C.F., Hemradj V.V., Hermanides R.S., Kedhi E., Ottervanger J.P., Zwolle Myocardial Infarction Study Group (2018). Coronary Artery Ectasia, an Independent Predictor of No-Reflow after Primary PCI for ST-Elevation Myocardial Infarction. Int. J. Cardiol..

[52] Roberts W.C. (2011). Natural History, Clinical Consequences, and Morphologic Features of Coronary Arterial Aneurysms in Adults. Am. J. Cardiol..

[53] Elahi M.M., Dhannapuneni R.V., Keal R. (2004). Giant Left Main Coronary Artery Aneurysm with Mitral Regurgitation. Heart.

[54] Sen F., Yılmaz S., Kuyumcu M.S., Ozeke O., Balcı M.M., Aydoğdu S. (2014). The Presence of Fragmented QRS on 12-Lead Electrocardiography in Patients with Coronary Artery Ectasia. Korean Circ. J..

[55] Saglam M., Karakaya O., Barutcu I., Esen A.M., Turkmen M., Kargin R., Esen O., Ozdemir N., Kaymaz C. (2007). Identifying Cardiovascular Risk Factors in a Patient Population with Coronary Artery Ectasia. Angiology.

[56] McCrindle B.W., Rowley A.H., Newburger J.W., Burns J.C., Bolger A.F., Gewitz M., Baker A.L., Jackson M.A., Takahashi M., Shah P.B. (2017). Diagnosis, Treatment, and Long-Term Management of Kawasaki Disease: A Scientific Statement for Health Professionals From the American Heart Association. Circulation.

[57] Thangathurai J., Kalashnikova M., Takahashi M., Shinbane J.S. (2021). Coronary Artery Aneurysm in Kawasaki Disease: Coronary CT Angiography through the Lens of Pathophysiology and Differential Diagnosis. Radiol. Cardiothorac. Imaging.

[58] Zou S., Hu B. (2025). Prevalence of IVIG Resistance in Kawasaki Disease: A Systematic Review and Meta-Analysis. Front. Pediatr..

[59] James K.E., Kalot M.A., Husainat N.M., Dua A.B., Byram K., Springer J.M., Lin Y.C., Turgunbaev M., Villa-Forte A., Gorelik M. (2021). Kawasaki Disease: A Systematic Review and Meta-Analysis of Benefits and Harms of Common Treatments. ACR Open Rheumatol..

[60] Díaz-Zamudio M., Bacilio-Pérez U., Herrera-Zarza M.C., Meave-González A., Alexanderson-Rosas E., Zambrana-Balta G.F., Kimura-Hayama E.T. (2009). Coronary Artery Aneurysms and Ectasia: Role of Coronary CT Angiography. Radiographics.

[61] Kim W.Y., Danias P.G., Stuber M., Flamm S.D., Plein S., Nagel E., Langerak S.E., Weber O.M., Pedersen E.M., Schmidt M. (2001). Coronary Magnetic Resonance Angiography for the Detection of Coronary Stenoses. N. Engl. J. Med..

[62] Zalewska-Adamiec M., Kuzma L., Bachorzewska-Gajewska H., Dobrzycki S. (2021). Fractional Flow Reserve in the Diagnosis of Ischemic Heart Disease in a Patient with Coronary Artery Ectasia. Diagnostics.

[63] Lee J.M., Choi K.H., Doh J.-H., Nam C.-W., Shin E.-S., Hoshino M., Murai T., Yonetsu T., Mejía-Rentería H., Kakuta T. (2020). Long-Term Patient Prognostication by Coronary Flow Reserve and Index of Microcirculatory Resistance: International Registry of Comprehensive Physiologic Assessment. Korean Circ. J..

[64] Bairey Merz C.N., Pepine C.J., Shimokawa H., Berry C. (2020). Treatment of Coronary Microvascular Dysfunction. Cardiovasc. Res..

[65] AHA Scientific Statement Update on Kawasaki Disease: Identifying and Managing Patients at High Risk. https://www.acc.org/latest-in-cardiology/articles/2025/04/28/16/36/aha-scientific-statement-update-on-kawasaki-disease.

[66] Saito N., Ebata R., Okunushi K., Yasukawa K., Hamada H. (2025). Treatment and Prognosis of Patients with Kawasaki Disease and Giant Coronary Artery Aneurysm: A Retrospective Observational Study. Cardiovasc. Diagn. Ther..

[67] Iwańczyk S., Smukowska-Gorynia A., Woźniak P., Grygier M., Lesiak M., Araszkiewicz A. (2022). Invasive Assessment of the Microvascular Coronary Circulation in Patients with Coronary Artery Aneurysmal Disease. Pol. Arch. Intern. Med..

[68] Antonopoulos A.S., Siasos G., Oikonomou E., Mourouzis K., Mavroudeas S.E., Papageorgiou N., Papaioannou S., Tsiamis E., Toutouzas K., Tousoulis D. (2016). Characterization of Vascular Phenotype in Patients with Coronary Artery Ectasia: The Role of Endothelial Dysfunction. Int. J. Cardiol..

[69] Sanidas E.A., Vavuranakis M., Papaioannou T.G., Kakadiaris I.A., Carlier S., Syros G., Dangas G., Stefanadis C. (2008). Study of Atheromatous Plaque Using Intravascular Ultrasound. Hell. J. Cardiol..

[70] Schoenhagen P., Nissen S.E., Tuzcu E.M. (2003). Coronary Arterial Remodeling: From Bench to Bedside. Curr. Atheroscler. Rep..

[71] Iwańczyk S., Lehmann T., Pławski A., Woźniak P., Hertel A., Araszkiewicz A., Stępień K., Krupka G., Grygier M., Lesiak M. (2024). Novel Genetic Variants Potentially Associated with the Pathogenesis of Coronary Artery Aneurysm: Whole-Exome Sequencing Analysis. Hell. J. Cardiol..

[72] Jone P.-N., Tremoulet A., Choueiter N., Dominguez S.R., Harahsheh A.S., Mitani Y., Zimmerman M., Lin M.-T., Friedman K.G., on behalf of the American Heart Association Rheumatic Fever, Endocarditis (2024). Update on Diagnosis and Management of Kawasaki Disease: A Scientific Statement from the American Heart Association. Circulation.

[73] Robertson T., Fisher L. (1987). Prognostic Significance of Coronary Artery Aneurysm and Ectasia in the Coronary Artery Surgery Study (CASS) Registry. Prog. Clin. Biol. Res..

[74] William J., Russell R., Nicholas T. (1983). Coronary Artery Surgery Study (CASS): A Randomized Trial of Coronary Artery Bypass Surgery. Circulation.

[75] Sánchez-Sánchez I., Cerrato E., Bollati M., Espejo-Paeres C., Nombela-Franco L., Alfonso-Rodríguez E., Camacho-Freire S.J., Villablanca P.A., Amat-Santos I.J., De la Torre Hernández J.M. (2024). Long-Term Prognosis of Coronary Aneurysms. JACC Cardiovasc. Interv..

[76] Johnson P.T., Fishman E.K. (2010). CT Angiography of Coronary Artery Aneurysms: Detection, Definition, Causes, and Treatment. Am. J. Roentgenol..

[77] Candreva A., Huwiler J., Gallo D., Schweiger V., Gilhofer T., Leone R., Würdinger M., Rizzini M.L., Chiastra C., Stehli J. (2025). Outcomes of Coronary Artery Aneurysms: Insights from the Coronary Artery Ectasia and Aneurysm Registry (CAESAR). Swiss Med. Wkly..

[78] Fujii T., Ikari Y. (2023). Clinical Outcomes of ST-Elevation Myocardial Infarction Patients Who Present Special Forms of ST-Segment Elevation. J. Electrocardiol..

[79] Arslan F., Núñez-Gil I.J., Rodríguez-Olivares R., Cerrato E., Bollati M., Nombela-Franco L., Terol B., Alfonso-Rodríguez E., Camacho Freire S.J., Villablanca P.A. (2022). Sex Differences in Treatment Strategy for Coronary Artery Aneurysms: Insights from the International Coronary Artery Aneurysm Registry. Neth. Heart J..

[80] Fan C.-H., Hao Y., Liu Y.-H., Li X.-L., Huang Z.-H., Luo Y., Li R.-L. (2020). Anti-Inflammatory Effects of Rosuvastatin Treatment on Coronary Artery Ectasia Patients of Different Age Groups. BMC Cardiovasc. Disord..

[81] Matta A., Campelo-Parada F., Nader V., Lhermusier T., Bouisset F., Blanco S., Roncalli J., Carrié D. (2023). Long-Term Outcomes of Conservative versus Invasive Approach of Coronary Aneurysm. Arch. Cardiovasc. Dis. Suppl..

[82] Khedr A., Neupane B., Proskuriakova E., Jada K., Kakieu Djossi S., Mostafa J.A. (2021). Pharmacologic Management of Coronary Artery Ectasia. Cureus.

[83] Parikh P., Banerjee K., Sammour Y., Ali A.F., Sankaramangalam K., Nair R., Ellis S., Raymond R., Tuzcu E.M., Kapadia S. (2019). Utilization and Outcomes of Polytetrafluoroethylene Covered Stents in Patients with Coronary Artery Perforation and Coronary Artery Aneurysm: Single Center 15-Year Experience. Catheter. Cardiovasc. Interv..

[84] Núñez-Gil I.J., Cerrato E., Bollati M. (2022). Stent-Grafts versus Drug-Eluting Stents in Arterial Aneurysms, Insights from the International Coronary Artery Aneurysm Registry (CAAR). REC Interv. Cardiol..

[85] Bikdeli B., Ujueta F., Rashedi S., Talasaz A.H., Kadakia K.T., Lopes R.D., White H.D., Steg P.G., Piazza G., Al-Lamee R.K. (2025). Antiplatelet and Anticoagulant Therapy in the 2025 ACC/AHA Guideline for Acute Coronary Syndromes. JACC.

[86] Esposito L., Di Maio M., Silverio A., Cancro F.P., Bellino M., Attisano T., Tarantino F.F., Esposito G., Vecchione C., Galasso G. (2022). Treatment and Outcome of Patients with Coronary Artery Ectasia: Current Evidence and Novel Opportunities for an Old Dilemma. Front. Cardiovasc. Med..

[87] Zhu X., Zhou Q., Tong S., Zhou Y. (2021). Challenges and Strategies in the Management of Coronary Artery Aneurysms. Hell. J. Cardiol..

[88] Amirpour A., Zavar R., Danesh M., Mirbod S.M., Zaker E., Moslemi F., Amini Z., Sadeghi M. (2024). Anticoagulant and Antiplatelet Treatment Effects on the Incidence of Major Cardiovascular Events in Patients with Coronary Artery Ectasia: An Updated Systematic Review. Indian Heart J..

[89] Araiza-Garaygordobil D., Gopar-Nieto R., Sierra-Lara Martínez D., Belderrain-Morales N., Sarabia-Chao V., Alfaro-Ponce D.L., Ontiveros-Mercado H., Mendoza-García S., Altamirano-Castillo A., Martinez-Amezcua P. (2022). Dual Antiplatelet Therapy Versus Antiplatelet Monotherapy Plus Oral Anticoagulation in Patients with Acute Coronary Syndrome and Coronary Artery Ectasia: Design and Rationale of OVER-TIME Randomized Clinical Trial. High Blood Press. Cardiovasc. Prev..

[90] D’Ascenzo F., Saglietto A., Ramakrishna H., Andreis A., Jiménez-Mazuecos J.M., Nombela-Franco L., Cerrato E., Liebetrau C., Alfonso-Rodríguez E., Bagur R. (2021). Usefulness of Oral Anticoagulation in Patients with Coronary Aneurysms: Insights from the CAAR Registry. Catheter. Cardiovasc. Interv..

[91] Gupta A., Seth A. (2021). Oral Anticoagulants for Coronary Artery Aneurysm: For Few or for All?. Catheter. Cardiovasc. Interv..

[92] Dummer K.B., Miyata K., Shimizu C., Tremoulet A.H., Gleason J., Gordon J.B., Burns J.C. (2023). DOACs in Patients With Giant Coronary Artery Aneurysms After Kawasaki Disease. JAMA Netw. Open.

[93] Ong S.K.A., Raj D., Lee A.W.X., Shen L., Poh K.K., Koh P.L., Quek S.C. (2026). Direct Oral Anticoagulants in Children with Giant Coronary Artery Aneurysms from Kawasaki Disease: A Systematic Review and Meta-Analysis. Front. Cardiovasc. Med..

[94] Ipek G., Gungor B., Karatas M.B., Onuk T., Keskin M., Tanik O., Hayiroglu M.I., Oz A., Borklu E.B., Bolca O. (2016). Risk Factors and Outcomes in Patients with Ectatic Infarct-Related Artery Who Underwent Primary Percutaneous Coronary Intervention after ST Elevated Myocardial Infarction. Catheter. Cardiovasc. Interv..

[95] Briguori C., Sarais C., Sivieri G., Takagi T., Di Mario C., Colombo A. (2002). Polytetrafluoroethylene-Covered Stent and Coronary Artery Aneurysms. Catheter. Cardiovasc. Interv..

[96] Khubber S., Chana R., Meenakshisundaram C., Dhaliwal K., Gad M., Kaur M., Banerjee K., Verma B.R., Shekhar S., Khan M.Z. (2021). Coronary Artery Aneurysms: Outcomes Following Medical, Percutaneous Interventional and Surgical Management. Open Heart.

[97] Zhu X., Wang Y., Zhu L., Zhou Q., Fan F. (2026). Surgical Repair of Multiple Coronary Artery Aneurysms with Multivessel Coronary Disease. JACC Case Rep..

[98] Tehrani S., Faircloth M., Chua T.-P., Rathore S. (2021). Percutaneous Coronary Intervention in Coronary Artery Aneurysms; Technical Aspects. Report of Case Series and Literature Review. Cardiovasc. Revasc Med..

[99] Viola L., Keita L., Veerasingam D. (2017). Surgical Treatment of a Giant Left Main Aneurysm. Interact. Cardiovasc. Thorac. Surg..

[100] Chura A.C., Arellano R.B., Quispe L.F., Rojas de la Cuba P., Chacalcaje K.C., Pachas G.L. (2025). Giant Left Main Coronary Artery Aneurysms Presenting as STEMI. JACC Case Rep..

[101] Will M., Kwok C.S., Nagaraja V., Potluri R., Weiss T.W., Mascherbauer J., Schwarz K. (2022). Outcomes of Patients Who Undergo Elective Covered Stent Treatment for Coronary Artery Aneurysms. Cardiovasc. Revascularization Med..

[102] Harnek J., James S., Lagerqvist B. (2019). Very Long-Term Outcome of Coronary Covered Stents: A Report from the SCAAR Registry. EuroIntervention.

[103] Hernández-Enríquez M., Belle L., Madiot H., Pansieri M., Souteyrand G., de Poli F., Piot C., Boueri Z., Gerbaud E., Boiffard E. (2021). Use and Outcomes of the PK Papyrus Covered Stent in France: SOS PK Papyrus Registry. Catheter. Cardiovasc. Interv..

[104] Naimi I., Morray B., Portman M.A., Steinberg Z.L. (2022). Use of the PK Papyrus Covered Coronary Stent in the Treatment of Kawasaki Disease-Associated Giant Coronary Artery Aneurysms. Catheter. Cardiovasc. Interv..

[105] Werner G.S., Ahmed W.H. (2017). Fenestration of a Papyrus PK Covered Stent to Recover the Occluded Left Main Bifurcation after Sealing a Left Main Perforation during a CTO Procedure. Cardiovasc. Revascularization Med..

[106] Iannopollo G., Ferlini M., Koziński M., Ormezzano M.F., Crimi G., Lanfranchi L., Camporotondo R., Visconti L.O., De Ferrari G.M., De Servi S. (2017). Patient Outcomes with STEMI Caused by Aneurysmal Coronary Artery Disease and Treated with Primary PCI. J. Am. Coll. Cardiol..

[107] Kim Y. (2024). Stent-Assisted Coil Embolization of Large Coronary Artery Aneurysm under Intravascular Ultrasound Guidance. Cardiol. J..

[108] Win H.K., Polsani V., Chang S.M., Kleiman N.S. (2012). Stent-Assisted Coil Embolization of a Large Fusiform Aneurysm of Proximal Anterior Descending Artery: Novel Treatment for Coronary Aneurysms. Circ. Cardiovasc. Interv..

[109] Geuns R.-J.v., Awad K., IJsselmuiden A., Koch K. (2014). The Role of Self-Expanding Stents in Patients with Atypical Coronary Anatomy. Interv. Cardiol. Rev..

[110] Lu H., Bekker R.J., Grundeken M.J., Woudstra P., Wykrzykowska J.J., Tijssen J.G.P., de Winter R.J., Koch K.T. (2018). Five-Year Clinical Follow-up of the STENTYS Self-Apposing Stent in Complex Coronary Anatomy: A Single-Centre Experience with Report of Specific Angiographic Indications. Neth. Heart J..

[111] Greiten L.E., Laan D., Joyce L.D., Greason K.L., Daly R.C., Schaff H.V., King K.S., Joyce D.L. (2020). Management of Coronary Artery Aneurysms at the Time of Surgical Revascularization. J. Surg. Res..

[112] Beckmann E., Rustum S., Marquardt S., Merz C., Shrestha M., Martens A., Haverich A., Ismail I. (2017). Surgical Treatment of Coronary Artery Aneurysms. J. Card. Surg..

[113] Gaudino M., Hameed I., Robinson N.B., Ruan Y., Rahouma M., Naik A., Weidenmann V., Demetres M., Tam D.Y., Hare D.L. (2021). Angiographic Patency of Coronary Artery Bypass Conduits: A Network Meta-Analysis of Randomized Trials. J. Am. Heart Assoc..

